# Altered Neurovascular Coupling in Subcortical Ischemic Vascular Disease

**DOI:** 10.3389/fnagi.2021.598365

**Published:** 2021-05-12

**Authors:** Xiaoshuang Liu, Runtian Cheng, Li Chen, Junwei Gong, Tianyou Luo, Fajin Lv

**Affiliations:** ^1^Department of Radiology, First Affiliated Hospital of Chongqing Medical University, Chongqing, China; ^2^Department of Radiology, Affiliated Hospital of North Sichuan Medical College, Sichuan, China

**Keywords:** subcortical ischemic vascular disease, neurovascular coupling, cerebral blood flow, functional magnetic resonance imaging, cognitive deficit, arterial spin labeling

## Abstract

Patients with subcortical ischemic vascular disease (SIVD) exhibit a high risk of cognitive impairment that might be caused by neurologic deficits and vascular injuries. However, the mechanism remains unknown. In current study, 24 normal controls (NC) and 54 SIVD patients, including 26 SIVD patients with no cognitive impairment (SIVD-NCI) and 28 SIVD patients with mild cognitive impairment (SIVD-MCI) underwent the resting-state functional MRI (rs-fMRI) and neuropsychological assessments. We combined regional homogeneity (ReHo) and cerebral blood flow (CBF) by using the global ReHo-CBF correlations coefficient and the ReHo/CBF ratio to detect the inner link between neuronal activity and vascular responses. Correlations between the ReHo/CBF ratio and neuropsychological assessments were explored in patients with SIVD. As a result, we identified significantly decreased global ReHo-CBF coupling in the SIVD-NCI group and SIVD- MCI group with respect to the NC. The SIVD-MCI group showed more serious decoupling of the global ReHo-CBF correlation. We also found a significantly abnormal ReHo/CBF ratio predominantly located in cognitive-related brain regions, including the left insula, right middle temporal gyrus, right precuneus, left precentral gyrus, and left inferior parietal lobule but not the supramarginal and angular gyri. The SIVD-MCI group showed more severe disorders of neurovascular coupling than the other two groups. Moreover, the ReHo/CBF ratio in the left precentral gyrus of the SIVD-NCI group exhibited a positive correlation with the MMSE scores. These findings suggested that patients with SIVD show abnormal neurovascular coupling at the early stage of the disease and during disease development. It might be associated with disease severity and cognitive impairment. Neurovascular decoupling in brain may be a possible neuropathological mechanism of SIVD.

## Introduction

Subcortical ischemic vascular disease (SIVD) is a devastating illness with unclear etiology. It is a leading cause of subcortical vascular cognitive impairment (SVCI) (Rosenberg et al., 2014). Inapparent or even non-existent cognitive impairment was presented in the early stage of SIVD, and such individuals may suffer a high risk of future cognitive deficits (Sachdev et al., 2014). It is important to explore the pathological changes in the early stage of the disease. As the most common type of cerebral small-vessel disease (CSVD), SIVD may be related to the damaged endothelial cells, abnormal perfusion, and disrupted structural and functional connections in the brain (Wallin et al., 2018; Thrippleton et al., 2019). These alterations can promote the dysregulation of the neurovascular unit (NVU) which comprised neurons, astrocytes, and vessels (Muoio et al., 2014). It is acknowledged that the NVU plays a vital role in maintaining the homeostasis of the cerebral microenvironment to support the normal function in the brain (Pasley and Freeman, 2008; Helman and Murphy, 2016). Under physiological condition, microvascular blood flow matches well with the neurons and glia within the NVU, which is called neurovascular coupling (Girouard and Iadecola, 2006). CSVD could disturb this coupling and lead to incongruities between the cerebral blood supply and neuronal activity which might be treated as the main cause of cognitive impairment, but the mechanisms still cannot be fully explained (Caruso et al., 2019; Moretti and Caruso, 2020).

In recent decades, neuroimaging techniques have provided evidence for neural impairment (Xu et al., 2018; Qin et al., 2019; van Leijsen et al., 2019) and vascular impairment (Wardlaw et al., 2017; Wong et al., 2019; Zhang et al., 2019) in patients with CSVD. As expected, several studies have revealed a close correlation between brain activity and cerebral perfusion, and the results confirmed that the impairment of any component of the NVU can affect the neurovascular coupling associated with SVCI (Zlokovic, 2010; Murphy et al., 2016). The functional connectivity (FC) of brain regions is widely studied by resting-state functional MRI (rs-fMRI) through testing blood oxygen level-dependent (BOLD) signals (Hirano and Yamada, 2013; Ma et al., 2020). It has been suggested to reflect the essential functional relevance in the brain (Fox and Raichle, 2007). Decreased FC in patients with SIVD has been found in specific regions involved in cognitive functional organizations (Li et al., 2014). Reduced efficiencies in cortical-subcortical circuits and disrupted pathways between distinct brain regions were found to be related to the cognitive dysfunctions (Reijmer et al., 2016; Liu et al., 2019b). Networks relevant to the cognitive function were also showed dysconnectivity in patients with SIVD (Liu et al., 2019a). Another analytical measure, the resting-state cerebral blood flow (CBF) is closely contacted with glucose utilization, oxygen consumption and aerobic glycolysis that maintaining cerebral activities (Vaishnavi et al., 2010). Arterial spin labeling (ASL) MRI is a non-invasive technique that can rapidly quantify CBF (Golay et al., 2004). Previous studies have revealed that the FC of brain regions showed similar patterns to the CBF (Liang et al., 2013; Zhu et al., 2013). Patients with SIVD suffer perfusion deficits accompanied by abnormal FC that may relate to the cognitive impairment (Sun et al., 2016).

However, these studies depended on only single imaging techniques that describe either the cerebral perfusion or neuronal activity, which could not comprehensively present the neurovascular coupling dysregulations in the disease. Neuronal activity and cerebral perfusion should be regarded as a functional complex. Combined BOLD and ASL technologies have been recently applied for studying neurovascular coupling because their correlations could be analyzed with the cooperativities of cerebral perfusion and neuronal activity of each voxel in the brain (Liang et al., 2013). Several studies combined the two parameters by evaluating their correlation coefficient and ratio to detect the alterations of NVU in diseases. The results have revealed the decoupling of NVU may be a crucial factor of the mechanisms related to cognitive impairment (Sheng et al., 2018; Hu et al., 2019; Stickland et al., 2019). It has been confirmed that CSVD could injure NVU and lead to SVCI (Shabir et al., 2018; Caruso et al., 2019). However, research rarely emphasizes the alterations of NVU in patients with SIVD by combining BOLD and ASL techniques.

The regional homogeneity (ReHo) is derived from BOLD signals that quantify the similarity of a given voxel to those of its nearest neighbors in a voxel-wise manner. Previous studies have shown that the ReHo of spontaneous activity is susceptible to SIVD which may correlate to the cognitive impairment (Liu et al., 2014; Diciotti et al., 2017). In addition, the CBF maps extracted from ASL signals are non-invasive measures that evaluate the cerebral perfusion. In the present study, we combined ReHo and CBF to detect the inner link between the neuronal activity and vascular response and its clinical significance in patients with SIVD. We hypothesized that patients with SIVD would show abnormal neurovascular coupling at the early stage of the disease and in disease development. The ReHo–CBF correlation coefficient measures the consistency of spatial distributions between cerebral blood supply and neuronal activity at the voxel level in whole brain, and the ReHo/CBF ratio represents the connectivity strength between neurons and the surrounding brain areas that are supplied by a unit of CBF; these were used to estimate regional dysregulations of neurovascular coupling. We expect to provide additional information on the neuropathological mechanisms of SIVD.

## Materials and Methods

### Participants

Seventy-eight participants were recruited for the study, including 24 normal controls (NC) and 54 patients with SIVD (26 patients with no cognitive impairment, SIVD-NCI; 28 patients with mild cognitive impairment, SIVD-MCI). Each subject signed an informed consent form approved by the ethics committee of first affiliated hospital of Chongqing medical university.

The criteria of patients with SIVD were listed as follows: (1) white matter hyperintensities: extended caps (>10 mm) or diffusely confluent hyperintensities with irregular shape (>25 mm) intruded into the periventricular and deep white matter; and (2) lacunar cases: at least two lacunas in deep gray matter and accompanied by moderate white matter hyperintensities. The SIVD-MCI patients would be recruited if they met the criteria of SIVD accompanied by cognitive deficits, including the subjective cognitive complaints and objective cognitive impairments, but not satisfying the Diagnostic and Statistical Manual of Mental Disorders, fifth edition (DSM-V). Meanwhile, the SIVD-MCI patients should also get the Clinical Dementia Rating Scale (CDR) scores of 0.5 and Mini-Mental State Examination (MMSE) scores ranging from 23 to 26. The SIVD-NCI patients were enrolled if they have met the diagnosis of SIVD with normal cognition and normal daily life abilities (defined as the CDR scores = 0 and MMSE scores ≥ 27). Exclusion criteria for all participants included (1) psychiatric disorders, such as schizophrenia or depression; (2) neurodegenerative disorder like Parkinson’s disease; and (3) anyone with a metallic foreign body or other relevant magnetic resonance (MR) scanning contraindications.

### Neuropsychological Assessment

All participants underwent the following neuropsychological assessments: (1) life abilities evaluation: Activities of Daily Living scale (ADL); (2) global cognition test: MMSE; (3) episodic memory test: Auditory Verbal Learning Test (AVLT); (4) language function test: Boston Naming Test (BNT); (5) visuospatial perception test: Clock Drawing Test (CDT); (6) executive functions and working memory assessment: Trail Making Test (TMT-A and TMT-B) and Stroop Test 1 and 2 (color-word); and (7) Hamilton Depression Scale (HAMD), which was used to exclude those with potential depression.

### MRI Acquisition

MRI scanning was performed on a GE Signa Hdxt 3.0 T scanner. rs-fMRI data was acquired using an echo-planar imaging (EPI) pulse sequence: repetition time (TR) = 2,000 ms; echo time (TE) = 40 ms; flip angle (FA) = 90°; slice thickness = 4 mm, no gap; field of view (FOV) = 240 × 240 mm^2^; matrix = 64 × 64; and timepoints = 240. A pseudo continuous ASL (pcASL) sequence with a 3D fast spin-echo acquisition and background suppression was applied for the perfusion imaging: TR = 5,216 ms; TE = 9.8 ms; spiral in readout of eight arms with 512 sample points; FOV = 240 × 240 mm^2^; post-label delay (PLD) = 2,525 ms; reconstruction matrix = 128 × 128; slice thickness = 4 mm, no gap; and number of excitations = 3. 3D-T1 weighted images were scanned as follows: TR = 8.3 ms; TE = 3.3 ms; flip angle = 15°; thickness = 1 mm, no gap; FOV = 240 × 240 mm^2^; matrix = 240 × 240, and voxel = 1 × 1 × 1 mm^3^. The scan parameters of the T2-FLAIR weighted images were scanned as follows: TR = 8,000 ms; TE = 26 ms; TI = 1,500 ms; thickness = 5 mm, no gap; FOV = 240 × 240 mm^2^; and matrix = 256 × 192.

### Data Preprocessing

fMRI data preprocessing was performed using the Data Processing Assistant for Resting-State fMRI (DPARSF)^1^ based on Statistical Parametric Mapping 8 (SPM8)^2^. The steps were listed as follows: (1) the first 10 timepoints were removed for each subject; (2) different acquisition times across slices were corrected by slice timing; (3) realignment for head motion was performed (head motion > 3.0 mm translation or >3.0° rotation were excluded). Instantaneous head motion of each volume was also evaluated by calculating the mean framewise displacement (FD), and no significant differences among three groups were found in the mean FD; (4) the linear trends from the image time series were removed; (5) filtering with a frequency range of 0.01–0.08 Hz was performed; (6) spatial normalization into the standard Montreal Neurological Institute (MNI) space was performed by using EPI templates and resampled to 3 × 3 × 3 mm^3^ voxels; and (7) the nuisance covariates like global mean signal, cerebrospinal fluid signal, white matter signal, and 24 head motion parameters were regressed.

The CBF maps were generated with the software provided by the scanner vendor, and the SPM8 software was used to normalize the CBF images into MNI space. The preprocessing steps were specifically involved as follows: (1) the individual CBF maps were non-linearly co-registered to MNI space which generated a customized CBF template; (2) the individual CBF maps were non-linearly co-registered to the customized CBF template; (3) non-brain tissue were removed from each co-registered CBF map and normalized by the subject’s global mean gray matter CBF value; and (4) the normalized CBF maps were smoothed by using a 6-mm full-width at half-maximum (FWHM) Gaussian kernel.

In addition, the white matter hyperintensity (WMH) volumes were assessed on the T2-FLAIR weighted images and 3D-T1 weighted images by AccuBrain^TM^, a brain quantification tool that calculates the brain structure and WMH volume in a fully automatic mode.

### Data Analysis

#### ReHo Calculation

ReHo data were computed by the DPARSF. Individual ReHo images were generated by calculating the Kendall’s coefficient of concordance (KCC) between the time series of each voxel and those of its 26 neighboring voxels within a gray matter mask (Zang et al., 2004). Then, each individual’s ReHo map was divided by its own global mean KCC value within the mask to achieve standardization. Finally, the standardized maps were then spatially smoothed with a 6 mm × 6 mm × 6 mm FWHM Gaussian kernel.

#### Coupling Analysis

Each subject underwent correlation coefficient analysis across voxels to quantitatively evaluate the consistency of spatial distributions between ReHo and CBF (Liang et al., 2013). The ratios of ReHo to CBF were computed by the DPARSF to evaluate the connectivity strength between neurons and the surrounding brain areas supplied by a unit of CBF. Meanwhile, the ratio of each voxel for each participant was transformed into a z-score to increase normality.

### Statistical Analysis

Demographics, WMH volumes, and the scores of neuropsychological assessments were compared using SPSS 23.0 software. Normality for continuous variables was examined using Skewness–Kurtosis test. One-way ANOVA and *post hoc* comparisons were used to evaluate statistical differences. The chi-square test and the Kruskal–Wallis *H* test were used to analyze the sex proportions and non-parametric data, respectively.

In ReHo–CBF correlation coefficient analysis and ReHo/CBF ratio analysis, ANCOVA and *post hoc* individual tests were used to compare the differences among three groups. Age, gender, and education were imported as covariates in statistical analysis to avoid any confounding effects. Multiple comparisons in ReHo/CBF ratio analysis were corrected using the cluster-level false discovery rate (FDR) method with a threshold of *P* < 0.05 after the initial cluster-forming threshold corresponding to *P* < 0.001. The same methods were applied to the ReHo and CBF data separately to detect significant differences among groups. The mean value of the ReHo/CBF ratio in each significant cluster was extracted and used for region of interest (ROI)-based analysis. The partial correlation analysis controlled for age, gender, and education was performed to find the relationships between the ReHo/CBF ratio values of significant ROIs and the neuropsychological assessments. *P* < 0.05 was set for statistical significance. Bonferroni correction for multiple testing was used to adjust the *P* values (*P*_*c*_ < 0.001).

## Results

### Demographics and Cognitive Characteristics

The demographics and WMH volumes are presented in Table 1. Compared with the SIVD-NCI group and NC group, the SIVD-MCI group showed a significantly higher WMH burden. The levels of systolic blood pressure and the total cholesterol in patient groups are significantly different from those in the NC group. No significant differences were found in age, gender, education, triglycerides levels, and diastolic blood pressure levels.

**TABLE 1:** Demographic and clinical characteristics of the participants.

	NC (*N* = 24)	SIVD-NCI (*N* = 26)	SIVD-MCI (*N* = 28)	*P* value
Gender (M/F)	10/14	16/10	16/12	0.147
Age (year)	68.43 ± 8.02	70.33 ± 3.83	70.73 ± 5.58	0.068
Education (year)	10.50 ± 2.59	10.37 ± 2.25	9.35 ± 1.68	0.117
Systolic BP (mmHg)	132.66 ± 16.61	143.23 ± 22.32^a^	149.79 ± 24.26^a^	0.026
Diastolic BP (mmHg)	80.37 ± 11.88	80.80 ± 11.84	79.37 ± 16.44	0.662
Triglycerides (mmol/L)	1.67 ± 1.91	1.43 ± 0.50	1.45 ± 0.71	0.357
Total cholesterol (mmol/L)	5.40 ± 1.26	4.89 ± 1.39^a^	4.69 ± 1.58^a^	0.041
WMH volume (ml)	1.46 ± 0.70	12.61 ± 5.0^a^	19.80 ± 8.80^a, b^	<0.001

*The data are presented as the means ± SDs. WMH, white matter hyperintensity. ^*a*^Significant compared to the NC group, *P* < 0.05. ^*b*^Significant compared to the SIVD-NCI group, *P* < 0.05.*

Table 2 presents the results of the neuropsychological assessments. The results of the SIVD-MCI group were significantly worse than those of the other two groups. When compared with the NC group, the SIVD-NCI group performed significantly worse on the TMT-B test, immediate recall test, and delay recall test of AVLT.

**TABLE 2:** Cognitive characteristics of the participants.

	NC	SIVD-NCI	SIVD-MCI	*P* value
MMSE	28.00 ± 1.06	27.85 ± 0.92	23.93 ± 1.90^a, b^	<0.001
CDR	0	0	0.5	/
ADL	20.03 ± 0.66	21.22 ± 0.81	23.87 ± 0.35^a, b^	<0.001
HAMD	2.45 ± 1.93	2.56 ± 1.49	2.46 ± 1.37	0.719
AVLT				
Immediate recall	9.54 ± 2.12	7.61 ± 2.73^a^	4.82 ± 2.53^a,b^	<0.001
Delay recall	8.83 ± 2.33	6.96 ± 3.05^a^	4.00 ± 2.69^a,b^	<0.001
Recognition	27.58 ± 1.67	27.50 ± 1.18	23.61 ± 4.45^a,b^	<0.001
BNT	24.00 ± 3.45	23.96 ± 3.36	18.36 ± 3.97^a,b^	<0.001
CDT	3.16 ± 0.70	3.46 ± 0.65	2.46 ± 1.10^a,b^	<0.001
TMT-A (s)	79.67 ± 25.72	104.30 ± 42.56	170.40 ± 72.03^a,b^	<0.001
TMT-B (s)	184.80 ± 80.33	265.12 ± 92.49^a^	391.00 ± 112.47^a,b^	<0.001
Stroop-1 test	107.87 ± 4.57	104.84 ± 6.96	81.94 ± 20.09^a,b^	<0.001
Stroop-2 test	99.91 ± 11.62	91.50 ± 13.68	67.57 ± 24.59^a,b^	<0.001

*^*a*^Significant compared to the NC group (*P* < 0.05). ^*b*^Significant compared to the SIVD-NCI group (*P* < 0.05). MMSE, Mini-Mental State Examination; HAMD, Hamilton Depression Rating Scale; AVLT, Auditory Verbal Learning Test; BNT, Boston Naming Test; CDT, Clock Drawing Test; TMT, Trail Making Test.*

### Global ReHo–CBF Coupling Analysis

**FIGURE 1:**
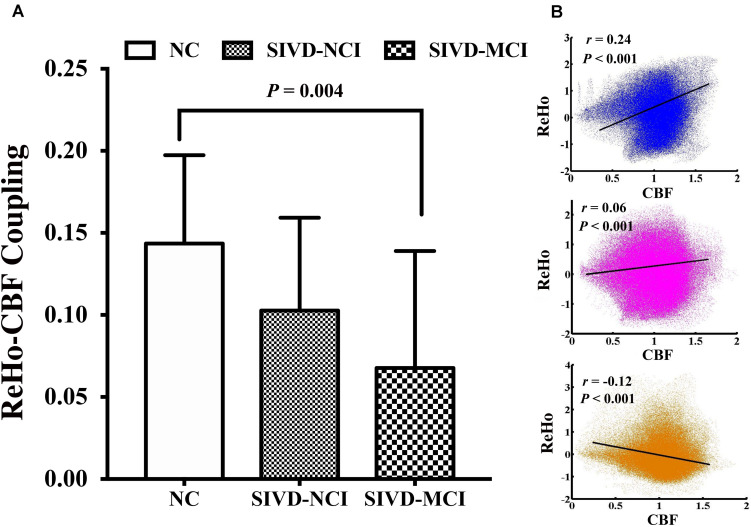
**(A)** Global ReHo–CBF coupling changes among three groups; SIVD-MCI group shows a significantly reduced ReHo–CBF coupling compared with NC group (*P* = 0.004). **(B)** Scatter plots of spatial correlation across voxels between ReHo and CBF of a normal control (blue; *r* = 0.24, *P* < 0.001); a SIVD-NCI patient (red; *r* = 0.06, *P* < 0.001) and a SIVD-MCI patient (orange; *r* = –0.12, *P* < 0.001).

Significantly across-voxel correlation coefficients between ReHo and CBF were found for each participant. Compared with NC, patients with SIVD showed a downward trend of ReHo–CBF coupling. The SIVD-MCI group had significantly more obvious ReHo–CBF decoupling than the NC group (*P* = 0.004) (Figure 1A). Three representative correlation maps of a NC participant, a SIVD-NCI patient and a SIVD-MCI patient are shown in Figure 1B. The NC showed a positive correlation between ReHo and CBF (blue; *r* = 0.24, *P* < 0.001), while the interaction was weaker in a SIVD-NCI patient (red; *r* = 0.06, *P* < 0.001). The ReHo values were inversely proportional to the CBF of a SIVD-MCI patient (orange; *r* = −0.12, *P* < 0.001).

### ReHo/CBF Ratio Analysis

**FIGURE 2:**
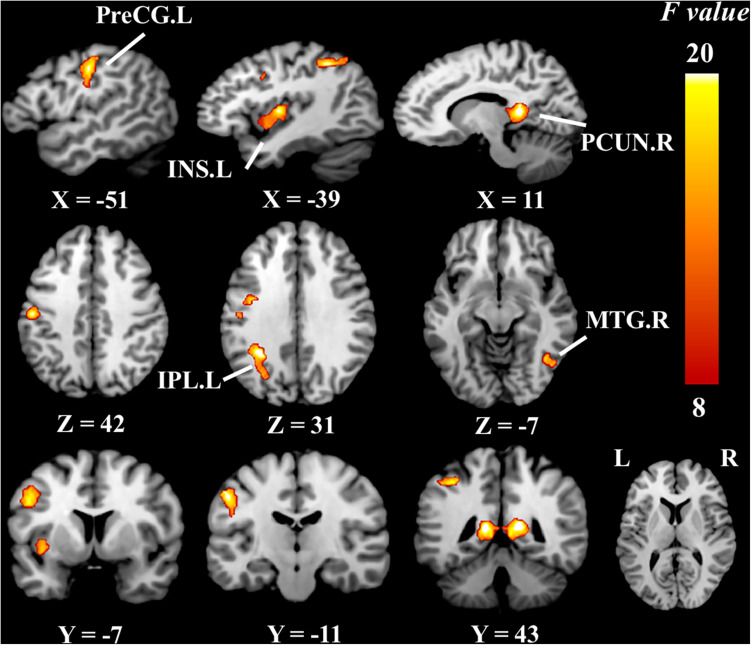
Brain regions of group differences in ReHo/CBF ratio values controlling for age gender and education (cluster-level *P* < 0.05, FDR corrected, cluster-forming threshold at voxel-level *P* < 0.001); *x*, *y*, *z*, coordinates of primary peak locations in MNI space; INS, insula; MTG, middle temporal gyrus; PCUN, precuneus; PreCG, precentral gyrus; IPL, inferior parietal, but supramarginal and angular gyri; L, left; R, right. The color bar represents the *F* values.

Brain regions with significantly altered ReHo/CBF ratios controlled for age, gender, and education (*P* < 0.05, cluster-level FDR corrected, cluster-forming threshold at voxel-level *P* < 0.001) were found in the left insula (INS.L), right middle temporal gyrus (MTG.R), right precuneus (PCUN.R), left precentral gyrus (PreCG.L), and left inferior parietal lobule but not the supramarginal and angular gyri (IPL.L) (Figure 2 and Table 3). We also detected significant differences in the values of ReHo and CBF separately. These differences are presented in Table 3.

**TABLE 3:** Group differences in the value of the ReHo/CBF ratio.

Brain region	MNI coordinates (mm)			Voxels	*F* value	Peak *T* value		
	*x*	*y*	*z*			SIVD-NCI vs. NC	SIVD-MCI vs. NC	SIVD-MCI vs. SIVD-NCI
INS.L	−36	−10	6	256	15.63		−5.59^a^	
MTG.R	64	−60	8	185	11.91	−4.67		
PCUN.R	10	−40	6	599	18.64		5.95^a, b^	
PreCG.L	−54	−14	44	599	16.28		−5.03^a^	−5.38
IPL.L	−32	−48	36	245	16.26		−5.53^a^	

*MNI, Montreal Neurological Institute; *x*, *y*, *z*, coordinates of primary peak locations in MNI space; INS, insula; MTG, middle temporal gyrus; PCUN, precuneus; PreCG, precentral gyrus; IPL, inferior parietal lobule but not the supramarginal and angular gyri; L, left; R, right; *P* < 0.05, cluster-level FDR corrected, cluster-forming threshold at voxel-level *P* < 0.001. ^*a*^Statistical differences in the value of ReHo. ^*b*^Statistical differences in the value of CBF.*

**FIGURE 3:**
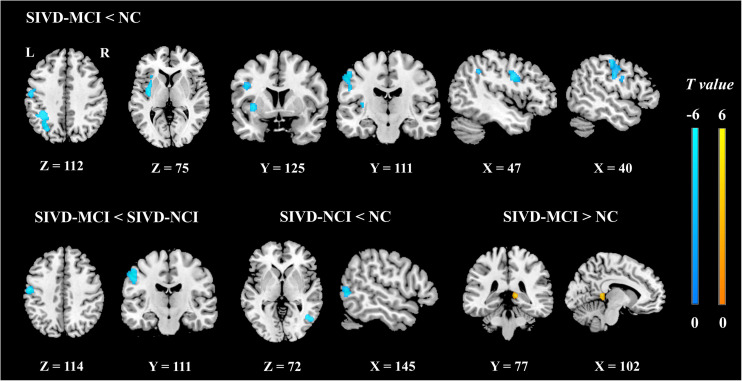
The *post hoc* analyses of ReHo/CBF ratio values between each pair of groups controlling for age, gender, and education (cluster-level *P* < 0.05, FDR corrected, cluster-forming threshold at voxel-level *P* < 0.001); *x*, *y*, *z*, coordinates of primary peak locations in the MNI space. The color bars represent the *T* values.

Figure 3 illustrates the *post hoc* results of the ReHo/CBF ratios between each pair of the groups. The ReHo/CBF ratios in the INS.L, PreCG.L and IPL.L of SIVD-MCI group and the ratios in the MTG.R of the SIVD-NCI group were significantly lower than those in the NC group. Compared with the SIVD-NCI group, the SIVD-MCI group showed a significantly decreased ReHo/CBF ratio at the PreCG.L. In addition, the increased ratio was found in the PCUN.R when compared between the SIVD-MCI group and the NC group (*P* < 0.05).

### Correlation Analysis

We found that the mean values of ReHo/CBF ratio at the PreCG.L in SIVD-NCI group exhibited a positive correlation with MMSE scores (*r* = 0.55, *P* = 0.006). However, the correlation did not persist after Bonferroni correction (*P*_*c*_ < 0.001).

## Discussion

In the current study, we evaluated the neurovascular coupling changes in SIVD patients by combining BOLD and ASL techniques. Although CBF was significantly correlated with ReHo in three groups, the SIVD patients had reduced global ReHo–CBF coupling relative to the NC group. Significantly altered ReHo/CBF ratios were found in the left insula, right middle temporal gyrus, right precuneus, left precentral gyrus, and left inferior parietal lobule but not the supramarginal and angular gyri. The ratio in the right middle temporal gyrus of the SIVD-NCI group was significantly different from that in the NC group. Additionally, we also found ReHo/CBF ratio deficits in the PreCG.L of the SIVD-NCI group were significantly correlated with the MMSE scores. These findings may improve our understanding about the neural mechanisms of SIVD from the perspective of dysfunction of the NVU.

It is well known that the NVU is the foundation of brain physiological activities based on neurons, astrocytes, smooth muscle cells, and endothelial cells (Nicolakakis et al., 2008). Coinciding with previous studies (Zhu et al., 2017; Guo et al., 2019), we found a significant correlation between CBF and ReHo in NC which represented normal neurovascular coupling that depends on the integrity of the NVU. Previous research has suggested that the endothelial cell damage could be an early pathological basis of neural injury in cerebral vascular disease (Wong et al., 2019). Injuries to the NVU could be the crucial point to cerebral vascular disease, but the mechanisms remain obscure (Murphy et al., 2016). In the present study, we found a weaker global ReHo–CBF coupling in a SIVD-NCI patient than that of a normal participant. Moreover, an inversed trend of coupling was found in a SIVD-MCI patient (Figure 1A). The results at the group level also showed decoupling of CBF and ReHo, and it may possibly illustrate that SIVD may weaken the connection between neuronal activities and the vascular responses and thus lead to dysfunction of the NVU (Figure 1B).

Compared to the global ReHo–CBF correlation coefficient, which describes the comprehensive changes of neurovascular coupling in the whole brain, the ReHo/CBF ratio could give more specific information about the alterations of NVU in local brain regions. In SIVD, endothelial distress in cerebral small vessels can potentiate flow dysregulation, chronic hypoxia, and stimulated inflammation in different brain areas (Arba et al., 2018; Moretti and Caruso, 2020). Hypoperfusion can further reduce the activities and damage the structure of microglia, astrocytes, and pericytes. Moreover, neural disorders can in turn affect the CBF and finally result in the dysfunction of NVU (Wardlaw et al., 2019).

Previous studies have confirmed that injuries to cerebrovascular endothelial cells and blood–brain barrier are essential for small-vessel disease (Nation et al., 2019; Wong et al., 2019). Destructions of the structural integrity of nerve fibers and abnormal FC of neurons in SIVD have been demonstrated as reliable biomarkers of human cognitive dysfunction (Liu et al., 2019a,b). However, a single index cannot reflect the interactions between the neuronal activity and cerebral perfusion, so the ReHo/CBF ratio may be used as a comprehensive marker to evaluate the NVU function. The ReHo/CBF ratio remains balanced in healthy brains, and deviation from this balance may result in either an increased or decreased ReHo/CBF ratio. We found that ReHo/CBF ratios in the left insula, left precentral gyrus, and left inferior parietal lobule but not the supramarginal and angular gyri of the SIVD-MCI group were significantly decreased, while the ReHo/CBF ratio in the right precuneus was increased with respect to those of the NC group. We also obtained the same statistical results by evaluating the ReHo and CBF values, respectively (Table 3). The decreased ratios may indicate reduced strength between neurons and the surrounding brain areas supplied by a unit of CBF, whereas explaining the increased ratio is slightly complicated because the ReHo and CBF values were both increased in the right precuneus. This may indicate that disorders of the NVU lead to abnormally enhanced functional connections supplied by redundant blood flow.

More importantly, we found that the ReHo/CBF ratio was significantly decreased in the right middle temporal gyrus of the SIVD-NCI group with respect to the NC group, and the ReHo/CBF ratio was also found significantly decreased in the left precentral gyrus of the SIVD-MCI group with respect to the SIVD-NCI group. However, the results did not show the significant intergroup differences in ReHo and CBF, respectively. This may indicate that the ReHo/CBF ratio could be more sensitive for detecting neurovascular coupling changes at the early stage of the disease and during its progression than the separate ReHo and CBF. In sum, CBF, ReHo, and the ReHo/CBF ratio could give comprehensive information and should be assessed jointly to explore pathological changes in SIVD.

In this study, we identified the altered neurovascular coupling in several brain regions associated with cognitive impairment. The SIVD-NCI patients showed a significantly decreased ReHo/CBF ratio in the left precentral gyrus, which was positively correlated with the MMSE scores. It is acknowledged that the precentral gyrus is the core brain region of the sensorimotor network and is mainly involved in cognitive control and motor activities (Park and Friston, 2013). This finding is consistent with the previous studies showing that the precentral gyrus is susceptible to cerebral vascular diseases, which may lead to cognitive injuries (Vipin et al., 2018; Caruso et al., 2019). Decreased ReHo/CBF ratios were also found in the insula, inferior parietal lobule, and middle temporal gyrus, which are regarded as the seats of specific cognitive domains. For example, the insula has been found involved in language processing (Gogolla, 2017), and hypoperfusion of this area is related to the degree of cognitive impairment (Sun et al., 2016). In our study, we found that SIVD patients performed worse in the Boston Naming Test than NC, which represented language function deficits. The results echo the previous study. The parietal regions and medial temporal regions have been suggested to be related to visuospatial attention and working memory (Wang et al., 2015; Lim et al., 2020). The lower scores in the Trial-Making Test and Stroop Test of SIVD patients in the present study may demonstrate that the abnormally decreased ReHo/CBF ratios in these brain regions were correlated with deficits in these specific cognitive domains. Unexpectedly, a significantly increased ReHo, CBF, and ReHo/CBF ratio were found in the precuneus, a pivotal node in regulating cognitive functions (van Leijsen et al., 2019). Abnormal deviation and activation of neurovascular coupling in the precuneus may be treated as a sensitive marker to reflect disturbances of the NVU associated with cognitive impairment.

The study has several limitations. Firstly, it is a relatively limited sample size research, and the severity of the disease was confined to the early stage. Patients with vascular dementia were not included. Future longitudinal studies with a larger group and dementia subjects are recommended. Secondly, technical limitations may have reduced the precision in the calculation of ReHo and CBF. Therefore, future studies with advanced methods and scanning parameters are recommended to strengthen the accuracy of the results. Thirdly, the correlation results between the ReHo/CBF ratio in the left precentral gyrus and the MMSE scores were not valid after Bonferroni correction. Thus, our findings should be regarded as an exploratory analysis. Finally, although the ReHo–CBF correlation and ReHo/CBF ratio are recommended for studying neurovascular coupling, they are both indirect measures for vascular response and neuronal activity. The ReHo–CBF correlation and ReHo/CBF ratio may not be sufficiently precise to truly reflect neurovascular coupling. This study has clarified a vital role of studying neurovascular coupling to understand the mechanisms of SIVD-related cognitive influence. Our research may provide a new particular imaging marker for deeply exploring the pathology of SIVD.

## Conclusion

In conclusion, we revealed a disrupted coupling between cerebral perfusion and functional activities of SIVD by jointly applying the BOLD and ASL techniques. Patients with SIVD show dysregulated neurovascular coupling that associate with disease severity and cognitive impairment. The findings may provide a new field in studying the neuropathological underpinning of the disease.

## Data Availability Statement

The raw data supporting the conclusions of this article will be made available by the authors, without undue reservation.

## Ethics Statement

The studies involving human participants were reviewed and approved by the Ethics Committee of The First Affiliated Hospital of Chongqing Medical University. The patients/participants provided their written informed consent to participate in this study.

## Author Contributions

TL, XL, and LC made substantial contributions to the conception and design of the study. XL, RC, and JG made substantial contributions to the acquisition of data. XL and LC made substantial contributions to the analysis of data. XL, XL, FL, and LC contributed to the interpretations of data. XL drafted the first version of the manuscript. All the authors revised the draft for intellectual content, gave their final approval of the final version for publication, and agreed to be accountable for all aspects of the work in ensuring that questions related to the accuracy or integrity of any part of the study are appropriately investigated and resolved.

## Conflict of Interest

The authors declare that the research was conducted in the absence of any commercial or financial relationships that could be construed as a potential conflict of interest.

## Footnotes

1 http://rfmri.org/DPARSF

2 http://www.fil.ion.ucl.ac.uk/spm
